# EMPLOYERS’ AGE-RELATED NORMS, STEREOTYPES, AND AGEIST PREFERENCES IN EMPLOYMENT

**DOI:** 10.1093/geroni/igz038.2109

**Published:** 2019-11-08

**Authors:** Jaap Oude Mulders

**Affiliations:** Netherlands Interdisciplinary Demographic Institute, The Hague, Netherlands

## Abstract

Social norms about retirement timing and stereotypes about qualities of younger and older workers are pervasive, but it is unclear how they relate to employers’ ageist preferences. Analyzing 2017 survey data from 960 Dutch employers, I study effects of employers’ retirement age norms and age-related stereotypes on their preferences for younger or older workers in three employment decisions: (1) hiring a new employee; (2) offering training; and (3) offering a permanent contract. Higher retirement age norms are related to lower preferences for younger workers in all employment decisions. More positive views about older workers’ soft qualities (such as reliability), but not about hard qualities (such as physical capacities), lead to managers being more favourable towards older workers for hiring and training, but not providing a permanent contract. The results show how ageist preferences of high-level organisational actors can influence outcomes in different employment decisions at the organisational level.

